# Job Satisfaction Mediates the Relationship between Psychosocial and Organization Factors and Mental Well-Being in Schoolteachers

**DOI:** 10.3390/ijerph20010593

**Published:** 2022-12-29

**Authors:** Vincenza Capone, Mohsen Joshanloo, Miriam Sang-Ah Park

**Affiliations:** 1 Department of Humanities, University of Naples Federico II, Via Porta di Massa 1, 80133 Naples, Italy; 2 Department of Psychology, Keimyung University, 1095 Dalgubeol Boulevard, Dalseo-gu, Daegu 42601, Republic of Korea; 3 Department of Psychology, Nottingham Trent University, Nottingham NG1 4FQ, UK

**Keywords:** teachers, job satisfaction, well-being, mental health, efficacy beliefs, organizational justice, school climate

## Abstract

The study aimed to explore the associations between the psychosocial and organizational factors, job satisfaction, and mental well-being of schoolteachers. A further aim was to examine whether job satisfaction mediated the relationship between these factors and mental well-being. A cross-sectional design was used to collect data using a self-reported questionnaire. Six hundred and nine schoolteachers were included in the final analyses. The relationships between the variables were tested in a path analysis model. The data fit the hypothesized model well. The results demonstrated that organizational justice, efficacy beliefs, and school climate were significantly associated with job satisfaction and that the relationships between these variables and mental well-being were mediated by job satisfaction. The findings have implications for programs targeted at promoting teachers’ well-being.

## 1. Introduction

Well-being is an important aspect of teachers’ mental health that is associated with positive personal and work outcomes. The happy–productive worker hypothesis, for instance, highlights the significance of worker well-being in job satisfaction and performance [1,2]. On the subject, some researchers have affirmed that mental well-being is a stronger correlate of job performance compared to job satisfaction [3]. In positive psychology [4], the role of positive factors, such as domains of life satisfaction and well-being, is emphasized and often studied in relation to psychological and job outcomes [5,6]. Life satisfaction (as a measure of well-being) was found to be positively associated with organizational commitment [7], lower turnover intentions [8,9,10], job self-efficacy [11], and organizational climate [12]. The literature emphasizes the importance of helping teachers to increase both their work well-being (e.g., job satisfaction) and mental well-being [13], which could have significant impacts on their work performance [14].

The role of psychosocial and organizational factors in predicting positive personal (i.e., mental health and well-being) and work outcomes (i.e., job satisfaction) has been highlighted. In the school context, teacher well-being is influenced by both individual and contextual factors [15], such as autonomy at work or the quality of relationships with superiors and with students [16]. However, in a recent systematic review of correlates of teacher well-being from various disciplines [17], the authors noted a high level of heterogeneity, not only in the definition of teacher well-being but also with regard to potential antecedents. Among the wide range of variables correlated with teachers’ well-being, they distinguished between ‘general’ and ‘job-related’ categories at the ‘individual’ or ‘contextual’ level and pointed out that although both objective and subjective aspects play a role in teachers’ well-being, the influence of subjective factors was greater. These results call for further data and investigation of well-being as a multidimensional construct, taking into account potential organizational and individual antecedents.

### 1.1. Efficacy Beliefs

In a recent review of teacher self-efficacy and its impact on well-being, Zee et al. [18] suggested that self-efficacy showed positive links with factors underlying teachers’ psychological well-being, including personal accomplishment, job satisfaction, and commitment. High levels of self-efficacy led to stronger commitment and involvement in day-to-day activities.

Bandura [19] affirmed that people’s perceptions of their competencies influenced their ability to cope with challenging and demanding conditions, which in turn would affect their psychological well-being. Teachers’ sense of efficacy was one of the most pivotal features that influenced both student motivational beliefs and teachers’ instructional quality. Teachers with a high level of self-efficacy showed more willingness to use advanced instructional methods [20], were more willing to work with struggling learners more persistently [21], were more enthusiastic about teaching [22], experienced less work burnout, and experienced lower degrees of stress and anxiety than instructors with low self-efficacy [23]. Studies about the role of teachers’ personal beliefs on their job satisfaction [24] suggest that teachers were more satisfied when they felt accomplished during their daily job at school. Personal efficacy beliefs have a main role in influencing professionals’ motivation and well-being [25]. Looking at the Italian context, Caprara et al. [11], in a study involving 2184 Italian high school teachers, reported a significant impact of teacher self-efficacy on their job satisfaction. A similar finding of the significant predictive power of teacher job satisfaction by teacher self-efficacy has also been reported in recent studies involving teachers of different cultures [15,26].

Teachers do not carry out their work alone but are part of a professional community that includes colleagues, the principal, and, albeit in a different way, students and families. So, teachers must manage their workload and personal/organizational resources in cooperation with colleagues in order to do a good job and feel satisfied. However, most of the research on efficacy beliefs has concentrated only on self-efficacy for determining life satisfaction. Only recently, in the school context, has collective efficacy seen conceptual, empirical, and methodological advancement. Teacher collective efficacy, which refers to the “perceptions of teachers in a school that the effort of the faculty as a whole will have a positive effect on students” [27] (p. 480), could be an important predictor of teacher effectiveness, and in determining teachers’ psychological, emotional, and social well-being [28]. In a recent study of Italian teachers working in secondary schools, Buonomo et al. [29] showed that collective efficacy was a significant predictor of job satisfaction and that the same was not true for self-efficacy [29]. Again, in an Italian study, teachers’ perceptions of personal and collective efficacy were correlated with both job satisfaction and mental well-being [30]. These results suggest the need to further investigate the relationship between personal and collective perceptions on the one hand and job satisfaction and context-free well-being on the other.

### 1.2. Organizational Justice

The working environment plays an important role in teachers’ well-being, especially when it comes to good relations and the feeling of being appreciated and fairly treated [29]. At the organizational level, distributive and procedural justice were significant in predicting employees’ subsequent personal satisfaction and commitment to the organization [31,32]. A study involving high school teachers found that organizational relationships characterized by the equity in the ratio of investments in the relationship with the school and outcomes from students were related to lower burnout levels [33].

The fair treatment of employees in the workplace leads to creating a high level of trust in an organization. Hoy and Tarter state that the development of trust in an institution can promote teamwork, the reduction of vulnerability, and problem-solving and encourages individuals to risk interdependence and leadership development [34]. All of the above enhance a principal’s ability to shape the mission and influence the behaviors of the institution’s members.

Organizational justice had an impact on teachers’ motivation and behavior [35] and had a significant total effect on subjective well-being [36]. A high level of organizational justice led to increased job satisfaction in the organization among employees and indicated more eagerness to achieve organizational objectives [37]. In the school context, teacher organizational justice was also positively associated also with emotional engagement, classroom connectedness, and individual functioning [38]. With regard to organizational justice in school settings, recently, attention has been paid to improving work environments, maintaining good relationships in work teams, and building a climate of trust [39]. Organizational justice has a significant overall effect on subjective well-being, and in a recent study, this relationship was fully mediated by collective efficacy [37]. This supports the importance of investigating perceptions of school justice simultaneously with other organizational variables and their role in teachers’ well-being.

### 1.3. School Climate

School climate refers to the quality and character of school life. School climate is based on patterns of students’, parents’, and school personnel’s experiences of school life and reflects norms, goals, values, interpersonal relationships, teaching and learning practices, and organizational structures. A socially supportive climate in the teaching profession seems to be a resource for teacher well-being, whereas conflicting peer relations among teachers seem to raise teacher burnout [40]. Further, emotional regulation and teachers’ stress levels were key factors affecting relationship quality in schools [41].

A school organizational climate reflecting higher levels of reflective dialogue was associated with both higher levels of collective teacher efficacy and self-efficacy [32]. A number of empirical studies have confirmed positive and significant effects between teacher job satisfaction and school climate factors, e.g., teacher collaboration, in improving student growth and learning outcomes [42]. In a more recent study, job satisfaction was found to be positively correlated with perceived school leadership styles, which are also a dimension of climate [43]. A study by Zakariya [44] showed a strong direct impact of school climate on job satisfaction and a mediating effect of teacher self-efficacy between school climate and job satisfaction, providing empirical evidence for the relations between teacher self-efficacy, job satisfaction, and school climate. These results are also confirmed by a recent work of Katsantonis [45] that found a significant impact of school climate on teachers’ self-efficacy and job satisfaction. With regard to well-being, teachers’ perceptions of school climate are an important area of focus when attempting to elucidate institutional factors associated with teacher mental health [46]. Literature has highlighted that perceptions of school climate were consistently related to negative mental health correlates, including work-related stress [47], but also to positive well-being [48].

Therefore, we expect school climate to play a role in the progressing mental health of teachers.

### 1.4. Job Satisfaction and Mental Well-Being

Hascher and Waber [17] highlighted teacher well-being in a multidisciplinary construct and suggested considering the specific working context of the teaching profession. On the basis of this evidence, for this study, we chose to consider not only occupational well-being but also context-free, considering both important for capturing the mental health dimension of teachers [48]. One of the main indicators of perceived well-being related to the work environment is job satisfaction.

This indicator refers to workers’ perception that their wants are attended to and reflects the amount of congruence between these perceptions and standards [49]. Locke [50] defined it as a pleasurable or positive emotional state resulting from the appraisal of one’s job or job experience. Skaalvik and Skaalvik [23] argued that teacher job satisfaction is the teachers’ affective reactions to their work and their teaching role. Implicit in these definitions is the importance of both affect and cognition. Thus, when people evaluate their jobs, both thinking and feeling are involved [51]. Studies have found job satisfaction to have significant positive relationships with happiness [52] and emotional, psychological, and social well-being [13,53,54]. Job satisfaction is influenced by workplace conditions [55], such as organizational justice [56] and school climate [57]. Recent studies have focused on the importance of a positive school environment in teacher well-being [58,59]. Hence, studies on schools have required approaching schools as holistic institutions and focusing on educationally-based mental health performance [60].

Job satisfaction has also been studied as an important mediator between working conditions/personality and work outcomes, such as job performance [61,62]. A systematic review and meta-analysis of 485 studies conducted by Faragher et al. [57] suggested that job satisfaction was an important factor influencing the health of workers. Considering positive workplace spillover effects, job satisfaction may enhance a range of well-being indicators [63]. In organizations that provided an environment that sufficiently attended to workers’ needs and demands and cared about their job satisfaction, employees had better mental well-being. Riasudeen et al. [59], for instance, highlighted that job satisfaction was a partial mediator between group cohesion and life satisfaction (as a measure of emotional well-being). Finally, demographic variables, such as age, gender, and level of experience, have also been found to be associated with job satisfaction and mental well-being in teachers [64,65].

### 1.5. Aim and Hypotheses

Although the literature suggests that factors like efficacy beliefs [66,67,68] and organizational-contextual factors (e.g., school climate, organizational justice) play a role in teacher well-being [34,69,70,71], the evidence is limited to work-centered perspectives that examine the independent contribution of job satisfaction to these variables. Personal and collective efficacy beliefs and the school climate appear to be particularly important aspects in the school context, where teamwork is a fundamental element of organizational functioning [72]. Furthermore, perceptions of organizational justice can also have a strong influence on job satisfaction and mental well-being [34]. Hence, the present study aimed to test the relationships between job satisfaction and mental well-being, exploring the associations among these variables and efficacy beliefs, organizational justice, and school climate while taking into account the effects of age and gender. 

Based on the previous findings, we aimed to examine whether job satisfaction mediated the relationship between the variables included in the study and mental well-being. We hypothesized that:

**Hypothesis 1** **(H1).** *Teachers’ job satisfaction and mental well-being would be positively related to efficacy beliefs, perceived school organizational climate, and organizational justice.*

**Hypothesis 2** **(H2).** *Efficacy beliefs, school climate, and organizational justice would be positively and indirectly related to mental well-being through job satisfaction as a mediator.*

## 2. Materials and Methods

### 2.1. Procedure

The participants in the study were teachers from Italian public schools. Schools were contacted by phone and asked to participate in the research, allowing a researcher access to the organization to administer a paper questionnaire. Forty (40) schools located in central, southern, and northern Italy were contacted. A total of 15 schools accepted: 8 from southern Italy, 3 from central Italy, and 4 from northern Italy. The questionnaires were self-completed, during working hours, in the presence of a researcher to answer any questions and address any doubts the participants might have. Teachers received an explanation of the objectives of the study and a guarantee of anonymity and confidentiality. The completion of the questionnaire took approximately 20–30 min. All but 44 participants agreed to complete the questionnaire. The study was conducted following the ethical guidelines of the APA.

### 2.2. Participants

A total of 609 teachers participated in this study. They were aged between 27 and 65 years (mean age 48.35 years; SD = 8.50). A total of 73.5% of the participants had more than 10 years of teaching experience (76.2% female). The gender distribution, although unbalanced in favor of females, is in line with Italian national statistics, which show that the majority of compulsory schoolteachers are female [73]. Most of the participants worked in high school (44.5%), 27.7% of the teachers worked in middle school, 16.1% in primary school, and 11.6% in kindergarten. All participants taught in schools with classes of 24 students on average. It should be noted that part of the data used in this study was used in a previous study [51] to investigate a different hypothesis.

### 2.3. Variables and Measures

Mental Well-being. The Italian Mental Health Continuum Short Form (MHC-SF) [66], containing 14 items, measures mental well-being as described in Keyes’ model [67]. The MHC–SF asks individuals how much of the time they functioned in a specific manner (e.g., “During the past month, how often did you feel that you belonged to a community”). Each item was rated on a 6-point response format, ranging from none of the time (0) to all of the time (5).

Job Satisfaction. The Job Satisfaction Scale [68] (Italian version by Magnavita et al.) [74] consists of 17 items measuring job satisfaction as a comprehensive feeling about the job and a related pattern of attitudes about various aspects of the job (e.g., “How satisfied are you with your principal?”). Each item was rated on a 7-point response format, ranging from very dissatisfied (1) to very satisfied (7).

Self-efficacy. The Teacher Self-Efficacy Scale [70] measures teachers’ beliefs in their ability to effectively manage a variety of tasks, commitments, and challenges related to their professional role (9 items, e.g., “How much can you do to get students to believe they can do well in school work?”). Each item was rated on a 7-point scale (ranging from nothing (1) to a great deal (7)). 

Collective efficacy. The School Collective Efficacy Scale [70] is composed of nine items measuring teachers’ beliefs about their school’s collective capabilities to influence student achievement, and it is based on teachers’ analysis of the teaching staff’s skills to effectively teach all students (e.g., “Our school always knows how to get out of trouble”). Teachers rated, on a 7-point scale, their agreement with each statement, from strongly disagree (1) to strongly agree (7).

School Climate. School climate was assessed using the three subscales of opportunities and school participation, school organizational relationships, and discipline from the Scholastic Situation Questionnaire—Teacher Version [71]. Opportunities and school participation (e.g., “In my school there is the possibility of using well-equipped laboratories”), as well as School organizational relationships (e.g., “The headmaster takes into account the opinion of the teachers in decision-making”), were each measured by ten items. School organizational relationships referred to the quality of relationships among teachers, principals, and students and gave information on the social and emotional climate of the school. Six items measured discipline (e.g., “Students respect the rules”), measuring the extent to which teachers and students respect the rules, as well as the frequency of incidents of violence. All items were scored on a 4-point frequency rating scale, ranging from completely false (1) to completely true (4).

Organizational Justice. The Italian version of the Organizational Justice Index [34,75] was used to measure teachers’ perceptions of fairness in the operation and administration of their schools. The scale contained 10 items (e.g., “The principal does not play favorites”). Respondents were asked to describe the behavior of teachers and administrators using a 6-point scale, ranging from strongly disagree (1) to strongly agree (6).

Internal consistencies for all the scales are reported in Table 1.

### 2.4. Statistical Analysis

We specified and tested path analysis models in Mplus 8.4 to investigate the relationships between our variables and to test our mediational hypotheses. To carry out the analysis, the Full Information Maximum Likelihood (FIML) estimator was applied. It can, in fact, accurately even handle a database that is missing data. We repeated the analyses with list deletion, and the results were very similar to those of FIML. Therefore, only the FIML results are reported here. Due to the 95% non-symmetric bootstrapping based on 10,000 replications for all parameters in our models, we calculated confidence intervals. In fact, the use of bootstrap confidence intervals is particularly recommended for testing hypotheses of indirect effects, as indirect effects are usually not normally distributed [72,73,76,77]. A minimum value of 0.95 for the Comparative Fit Index (CFI), a maximum value of 0.07 for the Root Mean Square Error of Approximation (RMSEA), and a maximum value of 0.08 for the Standardized Root Mean Square Residual (SRMR) were considered indicative of an acceptable fit [77]. In all analyses, sex (male = 0, female = 1) and age were included as covariates. School level was not a significant predictor of either mediators or outcomes in three ANOVAs, so we chose not to include it as a covariate in the mediation analyses.

## 3. Results

**Figure 1:**
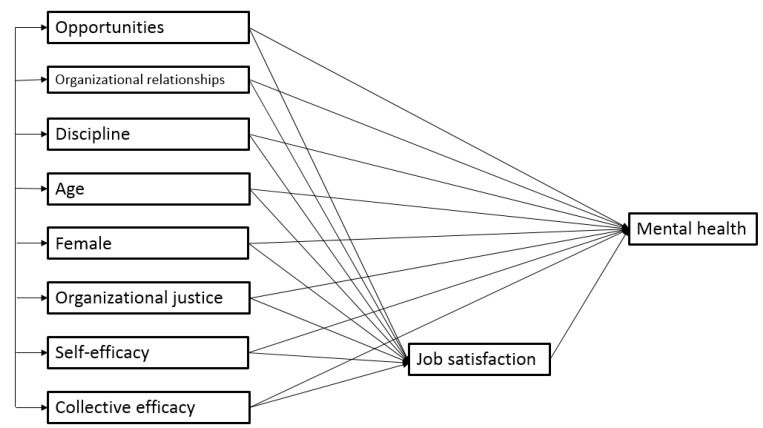
The saturated model.

Cronbach’s alphas and the inter-correlations among all of the variables are reported in Table 1. We started with a saturated model, as shown in Figure 1. A saturated or just-identified model is one in which each of the variables is related to all other variables in the model. This model reproduces the observed data and fits the data perfectly. To obtain an over-identified model whose fit to the data could be evaluated, the model was progressively simplified (trimmed) by removing non-significant directional paths one by one until there were no more non-significant paths in the model.

**Figure 2:**
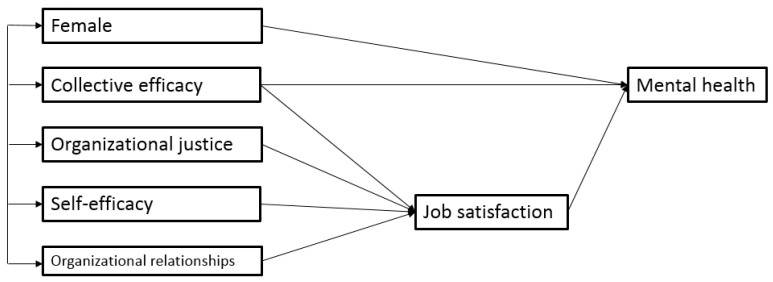
The final model.

The final model is shown in Figure 2. As can be seen, in addition to removing some paths, three variables (i.e., opportunities, discipline, and age) were removed in the final model because they turned out to be non-significant predictors of job satisfaction and mental health after accounting for the effects of other variables.

The fit indices for the final model were: χ^2^= 5.725 (df = 4, *p* = 0.220), SRMR = 0.011, RMSEA = 0.026 (90% CIs = 0.000, 0.070), CFI = 0.998, and TLI = 0.993. Based on the conventional standards used to evaluate model fit [77], these values indicate an excellent fit for our final model. The standardized estimates, *p* values, and bootstrap confidence intervals in the model are provided in Table 2. As can be seen, mental health was significantly predicted by job satisfaction, collective efficacy, and gender (R^2^ = 0.29), and organizational relationships, collective efficacy, self-efficacy, and organizational justice were significant predictors for job satisfaction (R^2^ = 0.57).

The final model contains the following four indirect paths to mental health:

1. Organizational relationships → job satisfaction → mental health
2. Collective efficacy → job satisfaction → mental health
3. Self-efficacy → job satisfaction → mental health
4. Organizational justice → job satisfaction → mental health.

These indirect-effect estimates and their confidence intervals are shown in Table 2. Given that the bootstrap confidence intervals for these estimates did not include zero, it can be concluded that all four mediational hypotheses were supported. In other words, it seems that job satisfaction functioned as a significant mediator between the four predictors (i.e., organizational relationships, collective efficacy, self-efficacy, and organizational justice) and mental health. The mediations for organizational relationships, self-efficacy, and organizational justice were full, considering that the direct effects from these predictors on mental health were non-significant (and thus removed).

However, for collective efficacy, the mediation was partial because the direct effect of this variable on mental health was significant. In sum, the present results suggest that organizational relationships, collective efficacy, self-efficacy, and organizational justice predict higher levels of mental health through their positive association with job satisfaction.

## 4. Discussion

The present study examined the associations among teachers’ efficacy beliefs, organizational factors, and mental well-being in a single model with job satisfaction as a mediator. Our contribution was aimed at applying justice theories to school contexts. We expected that the dimensions of school organizational climate, efficacy beliefs, and perceived organizational justice would predict the mental well-being of teachers partially or fully through job satisfaction. The results were consistent with our hypotheses. The mediation analysis showed that job satisfaction functioned as a significant mediator between organizational relationships, organizational justice, efficacy beliefs, and mental well-being. Organizational factors were also found to have significant impacts on teachers’ mental well-being. However, some of the variables were better predictors (i.e., had larger standardized effects) than others. Organizational justice was the best predictor of job satisfaction; meanwhile, collective efficacy was the weakest predictor (although both were significant). Kyriacou’s [78] review of the literature demonstrated that inequity was one of the sources of stress most frequently reported by teachers [79]. At the same time, several studies have shown that organizational justice was a critical factor in defining well-being in school [51].

In line with the prior empirical evidence, as well as our hypothesis, organizational justice was directly and significantly related to job satisfaction and indirectly to well-being. In Italy, in any school cycle, a group of students forms a “class” and stays together (with only a few variations due to contingent reasons) for the whole period of the cycle (in secondary schools, for 5 years), and the majority of the class teachers also remain stable for the same period. This particular condition creates a context where teachers’ and students’ interactions and communication are at the core of daily life in the school. So, increasing equity in the school setting could lead to positive outcomes not only for teachers but also for students and should be a priority for action at the organizational level [59]. Furthermore, as a sense of justice is a subjective feeling, teachers can adjust themselves cognitively to obtain such a sense. From the nature of the work, teachers should be aware that education is a kind of service labor and that some types of work input and performance are tangible, while many are intangible. Fostering such awareness and acceptance in teachers is especially important as it is difficult to achieve absolute fairness through the quantitative measurement of distribution standards.

As social cognitive theory has repeatedly emphasized, self-efficacy makes a difference in how individuals think, feel, and act, how environmental structures and stressors are perceived by people, and how they influence people’s goals, values, and behavior [17,80]. For teachers, this construct at high levels is positively associated with higher levels of job satisfaction [57]. Our results extend and improve recent work in this direction. Current literature suggests that beliefs about collective successful teaching experiences and working in a school with good policies may heighten teachers’ job satisfaction [48]. In the school context, the construct of efficacy was investigated as an emergent property of the organizational system, such as collective efficacy. Collective efficacy represents teachers’ beliefs that they, as a group/whole, can implement and organize courses of action affecting students and their levels of attainment. In the present study, the teachers’ collective efficacy was directly associated with job satisfaction. This result confirms previous studies on other cultural contexts addressing this association and, at the same time, the Italian educational system and its heterogeneity and complexity may have a role in explaining the role of collective belief on individual job satisfaction. Our results also highlighted the role of collective efficacy beliefs on mental well-being, supporting the hypothesis that the collective efficacy of teachers is related not only to the job sphere but also affects more global aspects. It is particularly interesting to note that the belief that the school, as a whole, can implement and organize courses of action can lead to improving students’ learning experience and their levels of attainment. Therefore, the present study demonstrates that collective efficacy could become a key parameter to recognize that leads to positive outcomes for both teachers and the quality of education. At the same time, the Italian educational system and its heterogeneity and complexity may have a role in explaining the role of collective belief in individual job satisfaction

School climate has been noted as one of the most important predictors of teacher job satisfaction [78]. This finding is suggestive of the necessary improvements that need to be made in order to promote job satisfaction through the school climate. A positive school climate also has the potential to contribute to teachers’ mental health and to provide an optimal environment to support student learning and growth. Knowledge of school climate and factors relating to teacher well-being are critical to allow for interventions to best support teachers and students in school settings [81]. In the present study, teachers’ successful dialogue with colleagues and other components of schools, such as perceived school climate, were found to be significantly associated with job satisfaction and also indirectly with mental well-being. This finding suggests that trainings on teamwork at school could improve teacher job satisfaction, as well as positively influence their perception of the school community and the profession in general.

Finally, consistent with prior research [82], our findings revealed that women displayed lower mental well-being than men. As argued in a previous study [34], one of the reasons for this result may be related to gender gap issues: maintaining a work-life balance may be more complicated and challenging for women, considering the traditional role of women in household management [83,84].

### 4.1. Limitations

Some limitations of the present study must be noted. Firstly, the results are mainly based on correlational analyses, and causality cannot be deduced. Future longitudinal studies are desirable, which will be able to investigate further the causal nature and stability of the investigated relationships. Furthermore, the data collection method based on self-report may carry common methodological biases. Future studies should attempt to assess these variables using independent sources of information. Although the sample is relatively large, we used a convenience sample, which is not representative of the population of Italian teachers. The type of recruitment did not allow us to obtain school-specific information in the context of multilevel analysis, again a factor that, if taken into account, can improve the quality of the work. Finally, our sample is not well balanced by gender, even though it is representative of the gender composition of Italian compulsory schoolteachers. Therefore, the results on gender effects are not sufficiently generalizable. In future studies, a larger and more representative sample should be used.

### 4.2. Implications

The results of this study have implications for improving teacher well-being and the quality of teaching and student learning experience. These results underline the role of job-related constructs, such as self-efficacy and job satisfaction, in affecting teachers’ psychosocial well-being. They are relevant for studies of mental well-being in specific work contexts and should be examined. It is also important to understand that well-being is a complex construct. Traditional teacher education programs tend to focus on the development of teacher knowledge and teaching techniques, discounting work-related aspects and relationship characteristics of teaching [85]. The findings from our present study may be helpful for designing and developing psychosocial intervention strategies.

In the education sector, our findings highlight the need to emphasize creating a positive and fair work environment for teachers [35]. Moreover, participation is a key activity for organizational functioning, no less so for the school. So, it will be important that each education system, and each school within the system, discusses which forces best fit their specific situation. Participation in organizational culture and decision-making could stimulate teachers to engage in various academic actions so that they feel or recognize that their contribution to the school is significant [86], with an important spin-off for students as well. The present study may be used as a starting point for discussion with teachers. Future research and interventions should focus on considering the factors investigated in the present study, particularly how to improve teachers’ job satisfaction.

The aforementioned recent review on teacher well-being has suggested that consensus needs to be found on the core elements of teacher well-being [17]. Affective and cognitive dimensions (e.g., enjoyment and satisfaction), positive and negative dimensions (e.g., satisfaction and worries), and psychological and physiological dimensions (mental and physical health) need to be integrated. From an applicative point of view, in terms of contributing to the debate on the topic, our work reinforces the idea of the multidimensionality of the construct of well-being, highlighting how job satisfaction and mental well-being should both be considered in an analysis of teachers’ health status.

## 5. Conclusions

Teachers must simultaneously balance instructional support, classroom management, planning and organization, and the facilitation of high-quality classroom relationships [81,87]. So, understanding teachers’ mental health is important not only for the aim of supporting teachers but also because these symptoms have implications for students [88]. Considering the results of the present study, schoolteachers must be provided with opportunities to develop and improve personal and organizational skills that could help them to feel more positive about their work. Research on the determinants of teacher mental well-being is vital for improving the school environment and interventions aimed at improving the quality of education [89]. The present findings suggest that scholars and practitioners, when referring to well-being, must differentiate between “well-being at work” and “context-free well-being,” improving both of them. Our results highlight the pivotal role of job satisfaction in teachers’ mental well-being. The findings also indicate that the relationships with job-related antecedents are stronger for job-related well-being, thus potentially offering a better understanding of how particular organizational factors could affect teachers’ well-being. Finally, regarding efficacy beliefs, a comprehensive understanding of the requirements of teaching and group dynamics would help pre-service and in-service teachers cultivate self- and collective efficacies in dealing with work demands. Harmonious relationships among teachers that lead to more positive collective identities can also facilitate better teaching and enhance their mental well-being. Collective efficacy and school climate have been found to be associated with mental health and, thus, with teachers’ subjective well-being [35]. Increased attention to these constructs in the school environment may even reduce absenteeism due to malaise, which is one of the psychological tensions that can signify a maladaptive response to stressors, one of which is a sense of organizational justice [29]. Furthermore, considering that the effects of organizational factors on teachers’ mental well-being may be highly dependent on their job satisfaction, teachers should be encouraged to develop a more constructive attitude toward their work and find proactive strategies to overcome the challenges they face at work. Although past studies evidenced the relationship of some work variables with job satisfaction, the present study extends the previous findings by identifying spillover effects on well-being. Teachers’ psychological well-being and social-emotional capacity are reported as key features that foster emotional and social learning activities (e.g., responsiveness, emotional support, and sensitivity) in the class [90]. In conclusion, our findings have important implications for ways to improve teacher well-being: they stress the importance of equipping teachers with personal/psychosocial skills to promote better well-being.

## Figures and Tables

**Table 1:** Descriptive statistics and correlations among measures.

	Alpha	Mean	SD	1	2	3	4	5	6	7	8
Mental health	0.89	2.90	0.94	1							
2. Job satisfaction	0.92	4.59	1.40	0.46 **	1						
3. Opportunities	0.78	2.18	1.41	0.28 **	0.48 **	1					
4. Organizational relationships	0.83	2.60	1.04	0.39 **	0.65 **	0.58 **	1				
5. Discipline	0.81	1.60	1.04	0.21 **	0.39 **	0.34 **	0.49 **	1			
6. Collective efficacy	0.90	4.46	1.00	0.41 **	0.37 **	0.20 **	0.33 **	0.27 **	1		
7. Self-efficacy	0.95	4.70	1.47	0.36 **	0.58 **	0.53 **	0.54 **	0.44 **	0.42 **	1	
8. Org. Justice	0.96	3.58	1.02	0.32 **	0.68 **	0.44 **	0.72 **	0.36 **	0.21 **	0.51 **	1
9. Age	–	48.35	8.50	−0.03	−0.02	−0.06	−0.05	0.08	0.02	0.04	−0.01

Note. ** *p* < 0.01.

**Table 2:** Standardized Estimates for Direct and Indirect Effects in the Final Model.

	Estimate	95% CI	
		Low	Up
Regression paths			
Job satisfaction → mental health	0.359 ***	0.278	0.436
Collective efficacy → mental health	0.272 ***	0.188	0.357
Female → mental health	−0.096 **	−0.166	−0.026
Organizational relationships → job satisfaction	0.193 **	0.089	0.305
Collective efficacy → job satisfaction	0.115 **	0.040	0.187
Self-efficacy → job satisfaction	0.221 ***	0.131	0.311
Organizational justice → job satisfaction	0.405 ***	0.304	0.499
*R* ^2^			
Mental health	0.289 ***	–	–
Job satisfaction	0.573 ***	–	–
Indirect effects			
1. Organizational relationships → job satisfaction → mental health	0.069 **	0.030	0.115
2. Collective efficacy → job satisfaction → mental health	0.041 **	0.014	0.070
3. Self-efficacy → job satisfaction → mental health	0.079 ***	0.043	0.120
4. Organizational justice → job satisfaction → mental health	0.145 ***	0.099	0.193
Total effects			
Collective efficacy → mental health	0.313 ***	0.232	0.393

Note: Sample size: 622; the number of bootstrap samples: 10,000; ** *p* < 0.01; *** *p* < 0.001.

## Data Availability

The data presented in this study are available on request from the corresponding author.

## References

[1] Zelenski J.M., Murphy S.A., Jenkins D.A. (2008). The Happy-Productive Worker Thesis Revisited. J. Happiness Stud..

[2] Taris T.W., Schreurs P.J.G. (2009). Well-Being and Organizational Performance: An Organizational-Level Test of the Happy-Productive Worker Hypothesis. Work Stress.

[3] Jones M.D. (2006). Which Is a Better Predictor of Job Performance: Job Satisfaction or Life Satisfaction. J. Behav. Appl. Manag..

[4] Seligman M.E.P., Csikszentmihalyi M. (2000). Positive Psychology: An Introduction. Am. Psychol..

[5] Czerw A. (2019). Diagnosing Well-Being in Work Context—Eudemonic Well-Being in the Workplace Questionnaire. Curr. Psychol..

[6] Özü Ö., Zepeda S., Ilgan A., Jimenez A.M., Ata A., Akram M. (2017). Teachers’ Psychological Well-Being: A Comparison among Teachers in U.S.A., Turkey and Pakistan. Int. J. Ment. Health Promot..

[7] Erdogan B., Bauer T.N., Truxillo D.M., Mansfield L.R. (2012). Whistle While You Work: A Review of the Life Satisfaction Literature. J. Manag..

[8] Lambert E.G., Kim B., Kelley T., Hogan N.L. (2013). The Association of Affective and Continuance Commitment with Correctional Staff Life Satisfaction. Soc. Sci. J..

[9] Rode J.C., Rehg M.T., Near J.P., Underhill J.R. (2007). The Effect of Work/Family Conflict on Intention to Quit: The Mediating Roles of Job and Life Satisfaction. Appl. Res. Qual. Life.

[10] Skaalvik E.M., Skaalvik S. (2017). Still Motivated to Teach? A Study of School Context Variables, Stress and Job Satisfaction among Teachers in Senior High School. Soc. Psychol. Educ..

[11] Caprara G.V., Steca P., Gerbino M., Paciello M., Vecchio G.M. (2006). Looking for Adolescents’ Well-Being: Self-Efficacy Beliefs as Determinants of Positive Thinking and Happiness. Epidemiol. Psichiatr. Soc..

[12] Cohrs J.C., Abele A.E., Dette D.E. (2006). Integrating Situational and Dispositional Determinants of Job Satisfaction: Findings from Three Samples of Professionals. J. Psychol..

[13] Capone V., Donizzetti A.R., Petrillo G. (2018). Classroom Relationships, Sense of Community, Perceptions of Justice, and Collective Efficacy for Students’ Social Well-Being. J. Community Psychol..

[14] Schaufeli W.B., Bakker A.B., Van Rhenen W. (2009). How Changes in Job Demands and Resources Predict Burnout, Work Engagement, and Sickness Absenteeism. J. Organ. Behav..

[15] You S., Kim A.Y., Lim S.A. (2017). Job Satisfaction among Secondary Teachers in Korea: Effects of Teachers’ Sense of Efficacy and School Culture. Educ. Manag. Adm. Leadersh..

[16] Billaudeau N., Alexander S., Magnard L., Temam S., Vercambre M.-N. (2022). What Levers to Promote Teachers’ Wellbeing during the COVID-19 Pandemic and Beyond: Lessons Learned from a 2021 Online Study in Six Countries. Int. J. Environ. Res. Public Health.

[17] Hascher T., Waber J. (2021). Teacher Well-Being: A Systematic Review of the Research Literature from the Year 2000–2019. Educ. Res. Rev..

[18] Zee M., Koomen H.M.Y. (2016). Teacher Self-Efficacy and Its Effects on Classroom Processes, Student Academic Adjustment, and Teacher Well-Being: A Synthesis of 40 Years of Research. Rev. Educ. Res..

[19] Bandura A. (1997). Self-Efficacy: The Exercise of Control.

[20] Haney J.J., Czerniak C.M., Lumpe A.T. (1996). Teacher Beliefs and Intentions Regarding the Implementation of Science Education Reform Strands. J. Res. Sci. Teach..

[21] Xiyun S., Fathi J., Shirbagi N., Mohammaddokht F. (2022). A Structural Model of Teacher Self-Efficacy, Emotion Regulation, and Psychological Wellbeing among English Teachers. Front. Psychol..

[22] Kunter M., Frenzel A., Nagy G., Baumert J., Pekrun R. (2011). Teacher Enthusiasm: Dimensionality and Context Specificity. Contemp. Educ. Psychol..

[23] Skaalvik E., Skaalvik S. (2010). Teacher Self-Efficacy and Teacher Burnout: A Study of Relations. Teaching and Teacher Education. Teach. Teach. Educ..

[24] Klassen R.M., Usher E.L., Bong M. (2010). Teachers’ Collective Efficacy, Job Satisfaction, and Job Stress in Cross-Cultural Context. J. Exp. Educ..

[25] Schwarzer R., Hallum S. (2008). Perceived Teacher Self-Efficacy as a Predictor of Job Stress and Burnout: Mediation Analyses. Appl. Psychol..

[26] Skaalvik E.M., Skaalvik S. (2014). Teacher Self-Efficacy and Perceived Autonomy: Relations with Teacher Engagement, Job Satisfaction, and Emotional Exhaustion. Psychol. Rep..

[27] Goddard R.D., Hoy W.K., Hoy A.W. (2000). Collective Teacher Efficacy: Its Meaning, Measure, and Impact on Student Achievement. Am. Educ. Res. J..

[28] Maslach C., Schaufeli W.B., Leiter M.P. (2001). Job Burnout. Annu. Rev. Psychol..

[29] Buonomo I., Fiorilli C., Benevene P. (2020). Unravelling Teacher Job Satisfaction: The Contribution of Collective Efficacy and Emotions towards Professional Role. Int. J. Environ. Res. Public Health.

[30] Petrillo G., Capone V. (2011). La Scala Di Efficacia Scolastica Nella Tutela Dei Diritti Dei Minori: Validazione Di Un Originale Strumento Di Rilevazione [The School Collective Efficacy Scale in the Protection of the Minor Rights: Validation of a New Scale]. Psicol. Scolastica.

[31] Meyer J.P., Smith C.A. (2000). HRM Practices and Organizational Commitment: Test of a Mediation Model. Can. J. Adm. Sci. Rev. Can. Sci. Adm..

[32] Fairchild S., Tobias R., Corcoran S., Djukic M., Kovner C., Noguera P. (2012). White and Black Teachers’ Job Satisfaction: Does Relational Demography Matter?. Urban Educ..

[33] Tadić M., Bakker A.B., Oerlemans W.G.M. (2013). Work Happiness among Teachers: A Day Reconstruction Study on the Role of Self-Concordance. J. Sch. Psychol..

[34] Hoy W.K., Tarter C.J. (2004). Organizational Justice in Schools: No Justice without Trust. Int. J. Educ. Manag..

[35] Capone V., Joshanloo M., Park M.S.-A. (2019). Burnout, Depression, Efficacy Beliefs, and Work-Related Variables among School Teachers. Int. J. Educ. Res..

[36] Weiss H.M., Cropanzano R. (1996). Affective Events Theory: A Theoretical Discussion of the Structure, Causes and Consequences of Affective Experiences at Work. Research in Organizational Behavior: An Annual Series of Analytical Essays and Critical Reviews.

[37] Herrera C., Torres-Vallejos J., Martínez-Líbano J., Rubio A., Céspedes C., Oyanedel J.C., Acuña E., Pedraza D. (2022). Perceived Collective School Efficacy Mediates the Organizational Justice Effect in Teachers’ Subjective Well-Being. Int. J. Environ. Res. Public Health.

[38] Mameli C., Biolcati R., Passini S., Mancini G. (2018). School Context and Subjective Distress: The Influence of Teacher Justice and School-Specific Well-Being on Adolescents’ Psychological Health. Sch. Psychol. Int..

[39] Carrasco A., Barraza D. (2020). La confianza y el cuidado en el liderazgo escolar de directoras chilenas. Calid. Educ..

[40] Bayarçelik E.B., Findikli M.A. (2016). The Mediating Effect of Job Satisfaction on the Relation between Organizational Justice Perception and Intention to Leave. Procedia Soc. Behav. Sci..

[41] Porru A., Dicataldo R., Leo I., Roch M., Lucangeli D. (2022). Back to School: Italian Teachers’ Perceptions of the Impact of COVID-19 on Personal and Social Well-Being and Teaching Methods. Int. J. Environ. Res. Public Health.

[42] Bottia M.C., Valentino L., Moller S., Mickelson R.A., Stearns E. (2016). Teacher Collaboration and Latinos/as’ Mathematics Achievement Trajectories. Am. J. Educ..

[43] García Torres D. (2019). Distributed Leadership, Professional Collaboration, and Teachers’ Job Satisfaction in U.S. Schools. Teach. Teach. Educ..

[44] Zakariya Y.F. (2020). Effects of School Climate and Teacher Self-Efficacy on Job Satisfaction of Mostly STEM Teachers: A Structural Multigroup Invariance Approach. Int. J. STEM Educ..

[45] Katsantonis I.G. (2020). Investigation of the Impact of School Climate and Teachers’ Self-Efficacy on Job Satisfaction: A Cross-Cultural Approach. Eur. J. Investig. Health Psychol. Educ..

[46] McLean L., Abry T., Taylor M., Jimenez M., Granger K. (2017). Teachers’ Mental Health and Perceptions of School Climate across the Transition from Training to Teaching. Teach. Teach. Educ..

[47] Skaalvik E.M., Skaalvik S. (2009). Does School Context Matter? Relations with Teacher Burnout and Job Satisfaction. Teach. Teach. Educ..

[48] Capone V., Petrillo G. (2020). Mental Health in Teachers: Relationships with Job Satisfaction, Efficacy Beliefs, Burnout and Depression. Curr. Psychol..

[49] Capone V., Petrillo G. (2015). Organizational Efficacy, Job Satisfaction and Well-Being: The Italian Adaptation and Validation of Bohn Organizational Efficacy Scale. J. Manag. Dev..

[50] Latham G.P. (2007). A Speculative Perspective on the Transfer of Behavioral Science Findings to the Workplace: “The Times They Are A-Changin”. Acad. Manag. J..

[51] Akinnola I.F., Oredein A.O. (2021). School Climate Indices as Predictors of Teacher Job Satisfaction and Performance in Oyo State, Nigeria. Teach. Educ. Curric. Stud..

[52] Noble T., McGrath H. (2008). The Positive Educational Practices Framework: A Tool for Facilitating the Work of Educational Psychologists in Promoting Pupil Wellbeing. Educ. Child Psychol..

[53] Shoshani A., Steinmetz S. (2014). Positive Psychology at School: A School-Based Intervention to Promote Adolescents’ Mental Health and Well-Being. J. Happiness Stud..

[54] Roffey S. (2012). Pupil Wellbeing—Teacher Wellbeing: Two Sides of the Same Coin?. Educ. Child Psychol..

[55] Hamama L., Ronen T., Shachar K., Rosenbaum M. (2013). Links between Stress, Positive and Negative Affect, and Life Satisfaction among Teachers in Special Education Schools. J. Happiness Stud..

[56] Lloyd T.J., Hastings R. (2009). Hope as a Psychological Resilience Factor in Mothers and Fathers of Children with Intellectual Disabilities. J. Intellect. Disabil. Res..

[57] Faragher E.B., Cass M., Cooper C.L. (2005). The Relationship between Job Satisfaction and Health: A Meta-Analysis. Occup. Environ. Med..

[58] Diener E., Seligman M.E.P. (2004). Beyond Money: Toward an Economy of Well-Being. Psychol. Sci. Public Interest.

[59] Riasudeen S., Singh P., Kannadhasan M. (2019). The Role of Job Satisfaction behind the Link between Group Cohesion, Collective Efficacy, and Life Satisfaction. Psychol. Stud..

[60] Lent R., Brown S. (2006). Integrating Person and Situation Perspectives on Work Satisfaction: A Social-Cognitive View. J. Vocat. Behav..

[61] Tschannen-Moran M., Hoy A.W., Hoy W.K. (1998). Teacher Efficacy: Its Meaning and Measure. Rev. Educ. Res..

[62] Buluc B., Gunes M. (2014). Relationship between Organizational Justice and Organizational Commitment in Primary Schools. Anthropologist.

[63] Parrello S., Ambrosetti A., Iorio I., Castelli L. (2019). School Burnout, Relational, and Organizational Factors. Front. Psychol..

[64] Skaalvik E.M., Skaalvik S. (2007). Dimensions of Teacher Self-Efficacy and Relations with Strain Factors, Perceived Collective Teacher Efficacy, and Teacher Burnout. J. Educ. Psychol..

[65] Eurostat Teachers in the EU. https://appsso.eurostat.ec.europa.eu/nui/submitViewTableAction.do.

[66] Petrillo G., Capone V., Caso D., Keyes C.L.M. (2015). The Mental Health Continuum–Short Form (MHC–SF) as a Measure of Well-Being in the Italian Context. Soc. Indic. Res..

[67] Keyes C.L.M., Wissing M., Potgieter J.P., Temane M., Kruger A., van Rooy S. (2008). Evaluation of the Mental Health Continuum–Short Form (MHC–SF) in Setswana-Speaking South Africans. Clin. Psychol. Psychother..

[68] Warr P., Cook J., Wall T. (1979). Scales for the Measurement of Some Work Attitudes and Aspects of Psychological Well-Being. J. Occup. Psychol..

[69] Magnavita N., Fileni A., Bergamaschi A. (2009). Satisfaction at Work among Radiologists. Radiol. Med..

[70] Borgogni L., Petitta L., Steca P. (2001). Personal and collective efficacy in organizational contexts. La Valutazione Dell’autoefficacia: Costrutti e Strumenti.

[71] Santinello M., Bertarelli P. (2014). School as Setting. Knowing the Community. The Analysis of the Environments of Daily Life.

[72] Bolin J.H., Hayes A.F. (2014). Introduction to Mediation, Moderation, and Conditional Process Analysis: A Regression-Based Approach. J. Educ. Meas..

[73] Jose P.E. (2013). Doing Statistical Mediation and Moderation.

[74] Magnavita N., Fileni A., Magnavita L., Mammi F., Roccia K., Matteis B., Colozza V., Vitale M. (2007). Job Satisfaction. Use of the Job Satisfaction Scale (JSS). G. Ital. Med. Lav. Ergon..

[75] Capone V., Petrillo G. (2016). Teachers’ Perceptions of Fairness, Well-Being and Burnout: A Contribution to the Validation of the Organizational Justice Index by Hoy and Tarter. Int. J. Educ. Manag..

[76] Asparouhov T., Muthén B. (2016). Structural Equation Models and Mixture Models with Continuous Nonnormal Skewed Distributions. Struct. Equ. Model. Multidiscip. J..

[77] Brown T.A. (2015). Confirmatory Factor Analysis for Applied Research.

[78] Kyriacou C. (2001). Teacher Stress: Directions for Future Research. Educ. Rev..

[79] Bentea C.-C. Relationships between Personality Characteristics and Attitude towards Work in School Teachers. Proceedings of the 6th International Conference Edu World 2014 Education Facing Contemporary World Issues.

[80] Srivastava A.P. (2017). Teachers’ Extra Role Behaviour: Relation with Self-Efficacy, Procedural Justice, Organisational Commitment and Support for Training. Int. J. Manag. Educ..

[81] Sibanda T., Sifelani I., Kwembeya M., Matsikure M., Songo S. (2022). Attitudes and perceptions of teachers toward mental health literacy: A case of Odzi High School, Mutare District, Zimbabwe. Front. Psychol..

[82] Van Horn J.E., Schaufeli W.B., Enzmann D. (1999). Teacher Burnout and Lack of Reciprocity. J. Appl. Soc. Psychol..

[83] Demir K. (2016). Relations between Teachers’ Organizational Justice Perceptions and Organizational Commitment and Job Satisfaction in the School: A Meta-Analysis. J. Hum. Sci..

[84] Lim S., Eo S. (2014). The Mediating Roles of Collective Teacher Efficacy in the Relations of Teachers’ Perceptions of School Organizational Climate to Their Burnout. Teach. Teach. Educ..

[85] Keyes C.L.M. (2002). The Mental Health Continuum: From Languishing to Flourishing in Life. J. Health Soc. Behav..

[86] Sutton R.E., Harper E., Saha L.J., Dworkin A.G. (2009). Teachers’ Emotion Regulation. International Handbook of Research on Teachers and Teaching.

[87] La Paro K.M., Pianta R.C., Stuhlman M. (2004). The Classroom Assessment Scoring System: Findings from the Prekindergarten Year. Elem. Sch. J..

[88] Gray C., Wilcox G., Nordstokke D. (2017). Teacher mental health, school climate, inclusive education and student learning: A review. Can. Psychol..

[89] De Carlo A., Girardi D., Falco A., Dal Corso L., Di Sipio A. (2019). When Does Work Interfere with Teachers’ Private Life? An Application of the Job Demands-Resources Model. Front. Psychol..

[90] Buettner C.K., Jeon L., Hur E., Garcia R.E. (2016). Teachers’ Social–Emotional Capacity: Factors Associated with Teachers’ Responsiveness and Professional Commitment. Early Educ. Dev..

